# Hypoxia alters the response of ovarian cancer cells to the mitomycin C drug

**DOI:** 10.3389/fcell.2025.1575134

**Published:** 2025-06-13

**Authors:** Aleksandra Gawrylak, Klaudia Brodaczewska, Roksana Iwanicka-Nowicka, Marta Koblowska, Agnieszka Synowiec, Lubomir Bodnar, Cezary Szczylik, Bogdan Lesyng, Rafał Stec, Claudine Kieda

**Affiliations:** ^1^ Military Institute of Medicine – National Research Institute, Laboratory of Molecular Oncology and Innovative Therapies, Szaserów, Warsaw, Poland; ^2^ Department of Immunology, Institute of Functional Biology and Ecology, Faculty of Biology, University of Warsaw, Warsaw, Poland; ^3^ Laboratory of Systems Biology, Faculty of Biology, University of Warsaw, Warsaw, Poland; ^4^ Laboratory of Microarray Analysis, Institute of Biochemistry and Biophysics, Polish Academy of Sciences, Warsaw, Poland; ^5^ Faculty of Medical and Health Sciences, Siedlce University of Natural Sciences and Humanities, Siedlce, Poland; ^6^ Department of Clinical Oncology and Radiotherapy, St. John Paul II Mazovia Regional Hospital in Siedlce, Siedlce, Poland; ^7^ Department of Oncology, Centre of Postgraduate Medical Education, European Health Centre, Otwock, Poland; ^8^ Division of Biophysics and Center for Machine Learning, Faculty of Physics, University of Warsaw, Warsaw, Poland; ^9^ Oncology Department, Medical University of Warsaw, Warsaw, Poland; ^10^ Center for Molecular Biophysics UPR 4301 CNRS, Orleans, France

**Keywords:** ovarian cancer, hypoxia, mitomycin c, transcriptome, differential gene expression, pathway analysis

## Abstract

**Introduction:**

Discrepancies between preclinical tests and clinical results raise serious concerns about the appropriateness of the current methodologies. In particular, cell biology approaches neglect fundamental physical parameters despite their relevance to *in vivo* conditions. Oxygen availability is critical for cell reactions; thus, the lack of consideration of hypoxia as the main regulator of the tumor microenvironment (TME) leads to misinterpreted data with consequences for translational applications. In this study, we show that mitomycin C (MMC), an antineoplastic antibiotic, is rarely used in ovarian cancer (OC) treatment despite its potential efficacy; we use MMC as an example of a treatment that warrants reevaluation under microenvironmental conditions, particularly during *in vitro* testing.

**Methods:**

To evaluate the effects of MMC and oxygen tension (pO_2_) on OC cells (SKOV3), HTA 2.0 microarrays were used, which demonstrated that hypoxia and MMC induced transcriptomic changes in OC cells. Their combination particularly emphasized the effect of pO_2_ modification on MMC activity. The most significant findings were verified in three other OC cell lines, namely, TOV112D, ES-2, and A2780.

**Results:**

Under normoxic conditions, MMC mostly affected several pathways associated with ribosome-related processes, whereas under hypoxic conditions, it induced modifications in the extracellular matrix (ECM). The most significantly upregulated gene in response to hypoxia–MMC treatment was *MMP1*, regulated by both MMC and hypoxia. Low pO_2_ levels during MMC treatment allowed the identification of important regulators, such as SPP1, and the corresponding processes, including cholesterol biosynthesis.

**Conclusion:**

Hypoxia modulated the effects of MMC on OC cells and identified genes that may serve as promising targets to enhance the effectiveness of MMC treatment.

## Introduction

Epithelial ovarian cancer (EOC), a global problem and challenge for modern oncology, affects vast numbers of women worldwide; in 2022, 324,398 new cases were recorded, reducing the life quality and length and causing numerous deaths (206,839) (Bray et al., 2024). Epidemiologically, the general downward trend in ovarian cancer (OC) morbidity results from progress in diagnostics and new targeted therapies. However, EOC still ranks eighth in terms of incidence and is the fifth most common cause of cancer-related death in women (Sung et al., 2021), thus being the most lethal tumor in the female reproductive system (Buklaho et al., 2023).

Predominant risk factors, such as hereditary mutations in genes predisposing to cancer development, account for 20%–25% of EOC cases (Bellcross, 2022). For years, OC treatment was based on a cytoreductive surgery and chemotherapy combination. The most active drugs for newly diagnosed advanced OC are platinum analogs and taxanes (Bogani et al., 2017; Pignata et al., 2019). Despite complete remission after such initial treatment, most patients (65%–80%) experience relapses within the first 5 years. The platinum-free interval is a predictor of treatment effectiveness (Duan et al., 2023). Maintenance therapy with PARP inhibitors and bevacizumab in the first, second, and subsequent lines of treatment is a recent progress in OC therapy (Sznurkowski, 2023).

In platinum-sensitive relapses, platinum salts remain the mainstay of treatment, while in platinum-resistant cases, progressing within 6 months (Colombo et al., 2019), no effective treatment exists. Mitomycin C (MMC), an alkylating cytostatic agent, was one of the drugs tested in platinum-resistant OC, showing a moderate 12% response (Hoskins et al., 1990). The response may be due to the inactivation of homologous recombination (HR) genes (Botrus et al., 2022). In tumors with functional repair deficiencies in HR, MMC may increase the activity of PARP inhibitors (veliparib) (Villalona-Calero et al., 2016). Chen X et al. showed that MMC may be particularly active in cancers with monoallelic and biallelic BRCA2 mutant genes. MMC significantly reduced BRCA2 mRNA and protein expression in BRCA2-mutated cell lines but not in wild-type BRCA2 cell lines, suggesting that BRCA2 inactivation is crucial for the MMC therapeutic effect (Chen et al., 2020).

Hence, the use of MMC in OC patients with HR disorders has gained attention. Promising effects were obtained in clinical trials of neoadjuvant treatment of OC combining MMC with cisplatin (Gorodnova et al., 2018) or in a three-drug regimen with doxorubicin (Gorodnova et al., 2021). MMC with cisplatin is also promising in treating recurrent OC caused by *BRCA1* gene mutations (Gorodnova et al., 2020). BRCA1 expression is dependent on oxygen tension (pO_2_) (Bindra et al., 2005; Lu et al., 2011; Fanale et al., 2013), with a strong impact on tumor suppressor factor activity (Li et al., 2018). OCs are highly hypoxia-dependent (Shih et al., 2021); thus, there is a need to study the drug performance under biologically representative conditions. Hypoxia—defined as the partial pressure of oxygen below the physiological levels—affects many cellular processes, including angiogenesis, epithelial-to-mesenchymal transition (EMT), and the acquisition of stem-like properties of aggressive cancer cells (Collet et al., 2012; Chouaib et al., 2018). Low oxygen tension stabilizes the hypoxia-inducible factor 1α (HIF-1α), and hypoxia reduces the effectiveness of drugs used to treat OC (Evans et al., 2012; Klemba et al., 2020).

Although MMC is used to treat several types of cancer (Lister-Sharp et al., 2000; Stec et al., 2014), knowledge about its effects on OC cells is limited (Strese et al., 2013). To date, its effects have not been studied under appropriate biologically relevant conditions, including hypoxia. To decipher its real effect in such conditions, a systemic approach was chosen using transcriptomic analysis to identify genes involved in the response of OC SKOV3 cells to MMC under hypoxic tumor-like conditions, and the most significant findings were verified in three other OC cell lines. Thus, this study aims to evaluate the effects of hypoxia, MMC, and their combination on SKOV3 cancer cells, with emphasis on transcriptomic analysis and the differential expression of genes.

## Materials and methods

### Cells and treatment procedure

Human OC cells SKOV3 (ATCC, Cat#HTB-77) were cultured in RPMI-1640 GlutaMAX^TM^ medium supplemented with 10% fetal bovine serum (FBS) (Thermo Fisher Scientific, United States; RPMI: Cat#61870036 and FBS: Cat#A5209401) for microarray experiments. SKOV3, TOV112D (ATCC, Cat#CRL-11731), ES-2 (ATCC, Cat#CRL-1978), and A2780 (ECACC, Cat#93112519) were maintained in Opti-MEM with 2% FBS (Thermo Fisher Scientific, United States; Opti-MEM: Cat#31985070 and FBS: Cat#A5209401) for other analyses. Mycoplasma-free (PromoKine, Cat#PK-CA91-1096) cells were passaged (p < 10) at 80% confluence using Accutase (BioLegend, United States, Cat#423201) for detachment.

SKOV3 cells seeded in a T-25 flask with 5 mL of medium were incubated for 24 h under normoxic conditions. After 24 h, the medium was replaced with preconditioned medium (incubated under normoxic and hypoxic conditions for 24 h before the experiment), with or without 1.16 μM of MMC (Sigma-Aldrich, Cat#M4287), and the flasks were incubated for the following 72 h before collecting materials (cells, protein, and RNA) for further analyses (FACS, WB, qPCR, ELISA, and microarrays). Cell numbers were assessed using the Trypan Blue exclusion test (Thermo Fisher Scientific, Cat#15250061) in a Burker chamber. Hypoxic conditions were achieved using an atmosphere of N_2_ 94%/CO_2_ 5%/O_2_ 1% (BioSpherix, Xvivo System Model X3).

The experimental setup combining the pO_2_ levels and MMC treatment is schematically presented in the following sections, resulting in four distinct experimental groups, namely, normoxia control (without treatment; N-C), normoxia with MMC treatment (N-MMC), hypoxia control (without treatment; H-C), and hypoxia with MMC treatment (H-MMC).


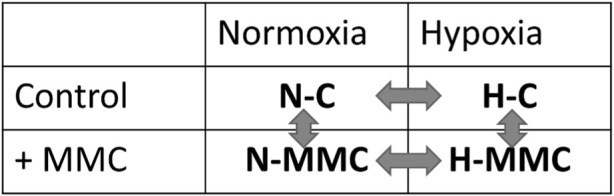


Detailed descriptions of the cell treatments used in the experiments reported in Supplementary Figures are provided in Supplementary Materials and Methods.

### Cell size, granularity, and cell cycle analysis

SKOV3 cells (2.25 × 10^5^ cells) seeded in T25 flasks and maintained under normoxia conditions were treated with MMC as mentioned above. Cell size and granularity were assessed by flow cytometry (FACSCalibur, Becton Dickinson, United States), using forward-scatter (FSC) and side-scatter (SSC) parameters, and data were analyzed using FCS Express 7 software (*De Novo* Software). Cell cycle analysis was performed by flow cytometry after 72 h of treatment; cells were fixed overnight at 4°C in 70% ethanol and stained with propidium iodide (BD Pharmingen™ PI Staining Solution, Becton Dickinson) to determine DNA content. Cell proportions in G0/G1, S, and G2/M phases were calculated using FCS Express 7 software (*De Novo* Software). A total of 10^4^ events were analyzed per sample.

### Evaluation of the Ki67+ population

The proportion of actively proliferating Ki67+ cells was investigated. Nuclear Ki67 is expressed in G1, S, G2, and M phases but absent in the G0 phase. Permeabilized cells were stained with a phycoerythrin-labeled (PE) anti-human Ki67 IgG1 antibody (Merck Millipore, United States, Cat#MCH100114) and analyzed by flow cytometry (BD FACSCalibur). IgG1-PE-stained cells served as the negative control. A total of 10^4^ events were analyzed per sample.

### LDH activity measurement

MMC cytotoxicity toward SKOV3 cells under normoxia vs hypoxia was assessed by measuring lactate dehydrogenase (LDH) activity in the SKOV3 cell culture supernatants after a 72-h treatment with (or without) MMC (LDH-Cytox Assay Kit, BioLegend, Cat#326401), and the orange formazan product was measured by absorbance at 490 nm using a microplate reader (Multiskan GO, Thermo Fisher Scientific Oy, Ratastie, Finland). Results are presented as relative toxicity.

### RNA isolation

Total RNA was extracted from the SKOV3 cells incubated with or without MMC under normoxia or hypoxia using a Direct-zol RNA Miniprep Kit (Zymo Research, Irvine, CA, United States, Cat#R2050). Genomic DNA was removed using a TURBO DNA-free Kit (Thermo Fisher Scientific, United States, Cat#AM 1907). RNA quality and concentration were determined by absorbance measurement at 230 nm, 260 nm, and 280 nm using the μDrop plate in a microplate reader (Thermo Fisher Scientific, Finland). RNA samples meeting the criteria of a 260/230 ratio >2 and a 260/280 ratio within 1.8–2.0 was used for further analysis.

### Microarray analysis

Total RNA isolated from SKOV3 cells was analyzed using Human Transcriptome Array (HTA) 2.0 (Affymetrix, Santa Clara, CA, United States, Cat#902162). RNA quality control (RNA integrity number, RIN) was assessed using the Bioanalyzer 2100 (Agilent Technologies, Waldbronn, Germany). HTA 2.0 arrays were performed as described by Koczan et al. (2018) and scanned using the GeneChip Scanner 3000 7G (Affymetrix).

HTA 2.0 microarray scans were visually inspected using Affymetrix GeneChip Command Console (AGCC), generating CEL files with processed pixel intensity values for each probe. The CEL files (N = 16, 1 GB of data) were imported to Transcriptome Analysis Console 4.0.2 software (TAC 4.0.2, Affymetrix) to summarize the probe set using the robust multi-array average (RMA) algorithm, including background correction, quantile normalization, and a log2 transformation of data, which was later used in the gene-level analysis. Each HTA 2.0 microarray contains data on 67,528 genes, 28,6263 full-length transcripts, and 67,0402 exons (including coding and non-coding transcripts).

### Transcriptomic data analysis

Differentially expressed genes (DEGs) were identified using TAC 4.0.2 software (Affymetrix, Santa Clara, CA, United States) and the ANOVA method for statistical testing with ebayes (Empirical Bayes Statistics for Differential Expression) correction for a small sample size. The DEG analysis was performed on the DEGs meeting the criteria of gene-level fold change < −2 or >2 and *p* < 0.05.

Venn diagram was prepared in InteractiVenn (Heberle et al., 2015) (https://www.interactivenn.net/). Gene Ontology (GO) and Kyoto Encyclopedia of Genes and Genomes (KEGG) pathway functional analyses were performed using SRplot software (https://www.bioinformatics.com.cn/en) and g:Profiler (Kolberg et al., 2023). DEGs without Entrez Gene ID were excluded from the analyses. GO terms were categorized into biological processes (BPs), cellular components (CCs), and molecular functions (MFs). Pathway analysis was also performed using Ingenuity Pathway Analysis (IPA, QIAGEN) software on DEGs that met the criteria of a gene-level fold change < −1.5 or >1.5 and a false discovery rate (*q-*value) < 0.05. DEGs used for the analyses are listed in Supplementary Table S1.

### Protein detection by Western blotting

Western blotting was performed as described previously (Brodaczewska et al., 2023). In brief, cells cultured in T25 flasks were detached with Accutase (BioLegend, United States, Cat#423201), washed twice with PBS, and lysed with RIPA buffer containing Cocktail inhibitors (Thermo Fisher Scientific, Waltham, MA, United States; RIPA: Cat#89901 and inhibitors: Cat#78430). Total protein concentration was assessed using the BCA assay (Thermo Fisher Scientific, Cat#23227), and a measure of 20 µg of total protein solubilized in the Laemmli sample buffer (Thermo Fisher Scientific, Alfa Aesar, Haverhill, MA, United States, Cat#J0015.AC) was loaded on 12% polyacrylamide gel. After transfer onto nitrocellulose membranes (Bio-Rad, Hercules, CA, United States, Cat#1620115), proteins were detected using Ponceau S staining (Thermo Fisher Scientific, Cat#A40000279). Non-specific binding was reduced by blocking (5% skimmed milk, 2 h; room temperature). Membranes were incubated overnight at 4°C in the solution of primary antibodies (anti-MMP1, R&D system, United States, Cat#MAB901), then washed with TBS-Tween, and incubated for 2 h at room temperature with the relevant secondary antibody conjugated with horseradish peroxidase (HRP) (Vector laboratories, United States, anti-Rabbit IgG: Cat#P1-1000, anti-Mouse IgG: Cat#P1-2000). Detection was performed using Luminol as an HRP substrate (Santa-Cruz, CA, United States) with X-ray films. Quantification of the integrated optical density (IOD) of the bands was calculated using ImageJ software and normalized to the IOD of loading control protein–vinculin (Santa-Cruz, CA, United States, Cat #sc-59803).

### Detection of secreted factors

Secreted VEGF, pro-MMP1, and total MMP1 were measured from SKOV3 cell culture supernatants after MMC treatment under normoxia and hypoxia using commercially available specific enzyme-linked immunosorbent assay kits (all DuoSet ELISA kits, R&D Systems, United States; VEGF: Cat#DY293B, pro-MMP1: Cat#DY900-05, and total MMP1: Cat#DY901B). Results were normalized to 10^6^ cells and calculated from the standard dose-response curve.

### Quantitative PCR

Two micrograms of RNA were reverse-transcribed into cDNA using Superscript IV (Thermo Fisher Scientific, Cat#18090050), according to the manufacturer’s protocol, and then subjected to real-time quantitative PCR (RT-qPCR) using Light Cycler 480 (Roche). Amplification conditions were 95°C (5 min), followed by 40 cycles at 95°C (10 s) and 60°C (30 s). The relative abundance of target mRNA was calculated according to the ΔΔ cycle threshold method (ΔΔCt). mRNA expression levels of PPIA and GUSB were used as internal controls to normalize each RT-qPCR reaction. The relative expression levels were calculated as fold enrichment of treated cells over control cells. The following TaqMan probes (Thermo Fisher Scientific) were used: MMP1 (Cat#Hs00899658_m1), VEGF (Cat#Hs00900055_m1), PPIA (Cat#Hs01565699_g1), and GUSB (Cat#Hs00939627_m1).

### Statistical analysis

Using GraphPad Prism v.10 software (GraphPad Software, San Diego, CA, United States), statistical tests were adjusted for each data set separately. The Shapiro–Wilk test was used to assess the normality of data distribution. For comparison between normoxia and hypoxia control and treated groups, two-way ANOVA followed by a Tukey’s *post hoc* test (or Kruskal–Wallis test with Dunn’s multiple comparison test for non-Gaussian distribution) was used. Data are expressed as the mean ± SEM for at least three independent biological experiments (n ≥ 3) and were considered statistically significant at p < 0.05.

## Results

### Mitomycin C cytotoxicity for SKOV3 cells in normoxia vs. hypoxia

**FIGURE 1:**
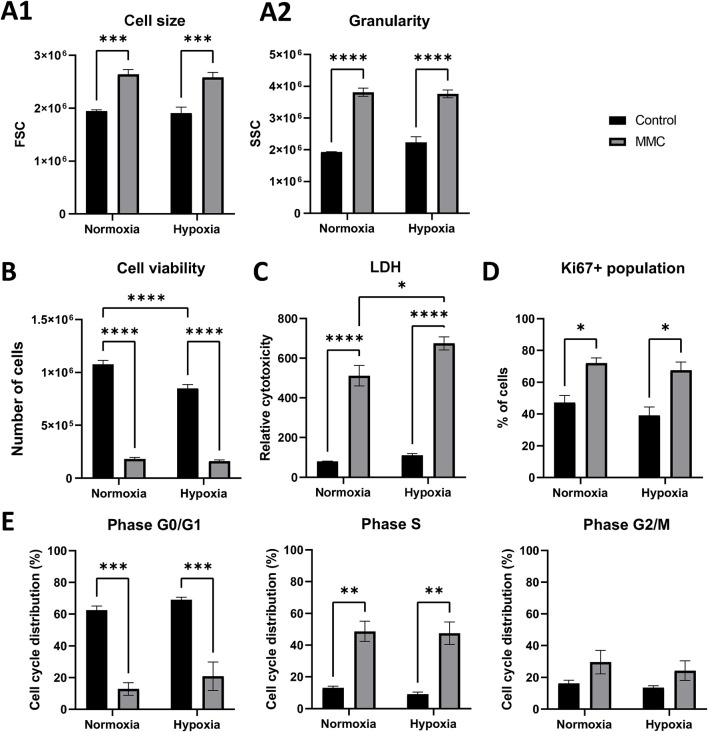
Influence of mitomycin C (MMC) on SKOV 3 cells under normoxic and hypoxic conditions. **(A1)** Cell size (FSC) and **(A2)** granularity (SSC) assessed by flow cytometry. **(B)** Quantification of number of SKOV3 cells. **(C)** Relative cytotoxicity of MMC assessed by the LDH assay. **(D)** Percentage of proliferative Ki67^+^ cells assessed by flow cytometry. **(E)** The cell cycle studied after propidium iodide (PI) incorporation and flow cytometry assessment: the percentages of cells among G0/G1, S, and G2/M phases of the cell cycle. **(A)** Detailed statistics description is provided in Supplementary Table S2.

To determine the effect of MMC on SKOV3 cell growth in normoxia and hypoxia, the morphology, cell number, LDH level, Ki67+ population, and cell cycle distribution were examined. Figures 1A1, A2 show cell morphology changes in both pO_2_-values; upon MMC treatment, cells were significantly bigger (*p* = 0.0005 and *p* = 0.0007 in normoxia and hypoxia, respectively) and more granular (*p* < 0.0001). Cell number was significantly reduced by drug treatment in both pO_2_-values (*p* < 0.0001; Figure 1B), and viability decreased under hypoxia (*p* < 0.0001). MMC was cytotoxic, as indicated by the elevated LDH level reflecting cell membrane damage under both normoxia and hypoxia conditions (*p* < 0.0001; Figure 1C). The MMC cytotoxic effect was significantly stronger under hypoxia, suggesting potential additive effects of MMC and low pO_2_ (*p* = 0.02; Figure 1C). However, MMC IC_50_ values did not significantly differ substantially upon pO_2_ changes (Supplementary Figure S1A). Exposure to MMC under normoxia (*p* = 0.0214) and hypoxia (*p* = 0.0101; Figure 1D) increased the Ki67+ cell proportion. Figure 1E shows the cell cycle distribution. In both pO_2_ conditions (*p* = 0.0006 and *p* = 0.0007), MMC reduced the percentage of SKOV3 cells in the G0/G1 phase and induced their accumulation in the S phase (*p* = 0.0036 in normoxia and *p* = 0.023 in hypoxia), similar to the Ki67+ cell distribution (Sun and Kaufman, 2018), without affecting the number of cells in the G2/M phases. Exemplary FACS histograms from cell cycle analysis are shown in Supplementary Figure S2.

### Mitomycin C and hypoxia alter the global gene expression of ovarian cancer cells

**FIGURE 2:**
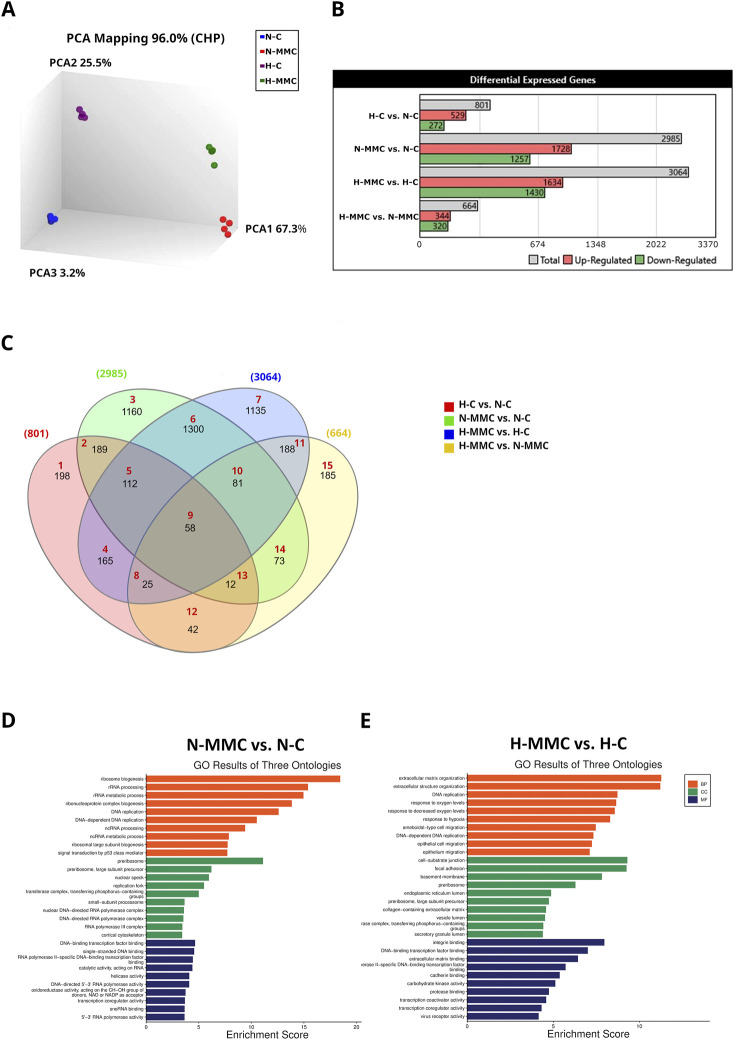
Global gene expression in SKOV3 cells treated with mitomycin C (MMC) under normoxia and hypoxia conditions. **(A)** Principal component analysis (PCA). **(B)** Differentially expressed gene analysis in SKOV cells using TAC 4.0.2; fold change < -2 or >2; p < 0.05. **(D)** Venn diagram–representing the overlapping groups of transcripts identified in **(B)**, red indicates different groups of genes mentioned in the article, black indicates the number of genes in a given group. N = 4. **(D,E)** The most significantly enriched Gene Ontologies (GO) in SKOV3 cells treated with MMC in hypoxia, based on molecular functions (MFs), biological processes (BPs), and cellular components (CCs). The enrichment score was calculated as −log (p-value). Fold change (FC) ≤ −2 and ≥2; p < 0.05; genes without Entrez Gene ID were excluded from the analysis. Analysis: **(D)** N-MMC vs N-C. **(E)** H-MMC vs. H-C.

As MMC IC_50_ on SKOV3 cells appeared similar under normoxia and hypoxia, we performed global transcriptome profiling to identify hypoxia-dependent pathways. Gene expression analysis was performed using HTA 2.0, which allows analysis at both the gene and exon levels. PCA analysis of HTA 2.0 data grouped samples into four separate sets, clearly indicating the proper group segregation (Figure 2A). PCA1 was responsible for explaining 67.3% of the variance—this dimension separated samples based on MMC treatment. PCA2 explained 25.5% of the variance—this dimension separated samples on the basis of pO_2_.

The DEGs were identified: a total of 801 genes were differentially expressed (529 up and 272 down) in hypoxic SKOV3 cells (compared to normoxic cells), 2,985 genes were differentially expressed (1728 up and 1,257 down) in MMC-treated SKOV3 cells in normoxia (compared to normoxic control cells), 3,064 genes were differentially expressed (1,634 up and 1,430 down) in MMC-treated hypoxic SKOV3 cells (compared to hypoxic control cells), and 664 genes were differentially regulated (344 up and 320 down) in hypoxic MMC-treated SKOV3 cells (compared to normoxic MMC-treated SKOV3 cells) (Figure 2B). The Venn diagram, in Figure 2C, presents the numbers of DEGs from all comparisons (H-C vs N-C, N-MMC vs N-C, H-MMC vs H-C, and H-MMC vs N-MMC). This identifies the commonly expressed (or mutually exclusive) genes for all comparisons. For example, N-C vs H-C comparison (gene group no. 1, red in the Venn diagram) identifies the genes differently expressed by hypoxic SKOV3 cells, mimicking the natural tumor environment. The Venn diagram indicates a group of genes modified in SKOV3 cells in both oxygen conditions (no. 6) and genes altered upon MMC treatment, distinctly reacting in normoxia (no. 3) as opposed to hypoxia (no. 7). This also identifies a group of genes (no. 15) that were altered upon MMC treatment but appeared distinguishable only upon comparison of the two pO_2_ conditions.

This initial analysis identified candidate genes that are the subject of further research and treatment. Genes modified under all investigated conditions are of particular interest (gene group no. 9); these 58 genes differentially expressed in all comparisons may, therefore, represent potential treatment targets. They are susceptible to MMC, regardless of the pO_2_ gradient in the tumor. Other alternatives for therapeutic options are 185 genes from group no. 15, which were differentially modulated between normoxia and hypoxia during MMC treatment, present potential therapeutic agents. These genes a) could not be identified as targets under normoxia conditions because they could only be identified as candidates in hypoxia and b) may represent exclusively effective targets because they are expressed under more physiologically relevant, hypoxic conditions.

### Mitomycin C treatment under normoxia affects ribosome biogenesis, whereas under hypoxia, it targets the extracellular matrix, as revealed by GO analysis

GO enrichment pathway analysis (Figure 2D) of DEGs identified in MMC-treated SKOV3 cells in normoxia showed the most significantly affected BPs being connected with ribosome biogenesis (GO:0042254) and rRNA processing (GO:0006364). The most affected CC was the preribosomal component (GO:0030684). The most affected MF was connected with “DNA-binding transcription factors binding” (GO:00140297). When treatment was performed under hypoxia (Figure 2E), the most significantly altered BPs were related to the extracellular matrix and structure organization (GO:0030198 and GO:0043062, respectively), while the most affected CCs concerned cell–substrate junction (GO:0030055) and focal adhesion (GO:0005925). The most affected MFs were related to integrin binding (GO:0005178) and DNA-binding transcription factor binding (GO:00140297). The action of hypoxia was also confirmed by GO analysis (Supplementary Figure S3).

### Mitomycin C-induced senescence is impaired by hypoxia

**FIGURE 3:**
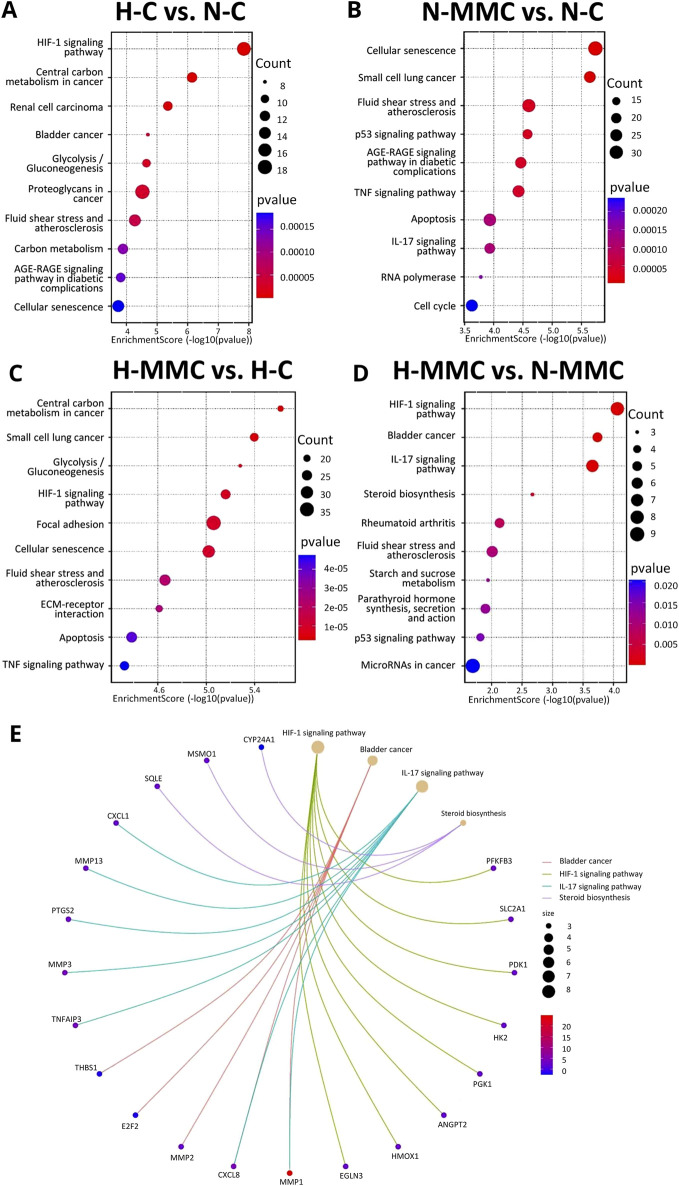
Pathways altered in all DEGs identified in SKOV3 cells under the influence of hypoxia and mitomycin C (MMC) based on the KEGG database. **(A)** Effect of hypoxia (H-C vs N-C). **(B)** Effect of MMC in normoxia (N-MMC vs. N-C). **(C)** Effect of MMC in hypoxia (H-MMC vs. H-C). **(D)** Effect of pO_2_ on MMC treatment (H-MMC vs. N-MMC). Fold change (FC) ≥ 2; *p <* 0.05; genes without Entrez Gene ID were excluded from the analysis. **(E)** C-net plot of pathways/processes altered in SKOV3 cells treated with MMC in hypoxia (vs. N-MMC), based on the KEGG database. Fold change (FC) ≤ −2 and ≥2; p < 0.05; genes without Entrez Gene ID were excluded from the analysis.

**FIGURE 4:**
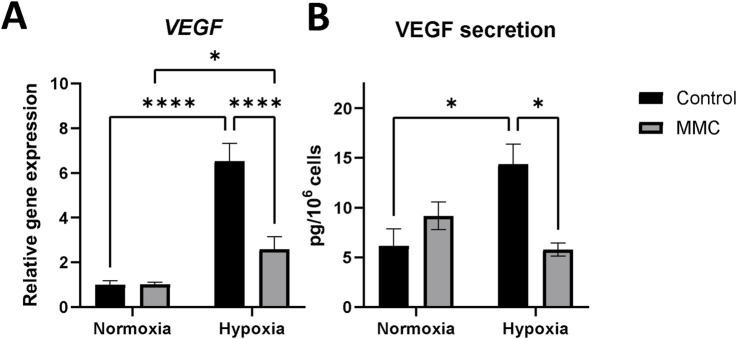
Expression of VEGF on gene and protein levels in SKOV3 cells treated with mitomycin C (MMC) in normoxia and hypoxia. **(A)** The *VEGF* mRNA expression level was measured by RT-qPCR and normalized to *PPIA* and *GUSB* expressions and normoxia control as 1. **(B)** Level of VEGF secretion in the cell culture supernatant measured by ELISA; results were normalized to normoxia control set as 1. A detailed statistics description is provided in the Supplementary Table S2.

To further differentiate the drug response by normoxic and hypoxic cells, KEGG pathway analysis was performed. It showed that HIF-1 signaling was the most significantly altered pathway in control hypoxic SKOV3 cells (Figure 3A: H-C vs N-C), as confirmed by the upregulation of the VEGF gene and protein expression (H-C vs N-C: mRNA, *p* < 0.0001; secretion, *p* = 0.0218, Figures 4A,B).

Cellular senescence is the most significantly altered process identified by KEGG analysis in normoxic SKOV3 cells after MMC treatment (Figure 3B). When the drug is added under hypoxic conditions, other pathways/processes become more important, including those related to central carbon metabolism in cancer, small-cell lung cancer, glycolysis/gluconeogenesis, HIF-1 signaling, and focal adhesion (Figure 3C). Moreover, the expression levels of p-S6 and p21 proteins were significantly upregulated upon MMC treatment under normoxia conditions (H-C vs N-C: for p-S6, *p* = 0.0323; for p21, *p* = 0.0019), while under hypoxia conditions, the upregulation was also significant for p21 (*p* = 0.0009) and p-S6 (*p* = 0.1627) (Supplementary Figure S4A-C). Correspondingly, SKOV3 cell size and granularity were significantly increased, suggesting the induction of senescence in response to MMC (Figure 1A1, A2). A total of 22 senescence-related genes were commonly changed in both pO_2_-values, while another 12 genes changed only under normoxia (N-MMC vs N-C) and 8 genes only under hypoxia (H-MMC vs. H-C) (listed in Supplementary Figure S5), suggesting that senescence induction involves different pathways depending on pO_2_. According to the GO analysis via g:Profiler, the first group is associated with cell division (GO:0051301), and the second group is associated with adenine transport (GO:0015853), indicating that hypoxia significantly modulates the SKOV3 cell response to the drug.

### MMP1 is the most upregulated gene from the IL-17 signaling pathway

The KEGG analysis revealed differential effects of pO_2_ on MMC-treated SKOV3 cells, highlighting the HIF-1 signaling pathway (hsa04066, Figure 3D), bladder cancer-related processes (hsa05219, Figure 3D), and the IL-17 signaling pathway (hsa04657, Figures 3D,E). *MMP1*, the most upregulated gene in this pathway, changed in all four comparisons, as first shown by the Venn diagram (Figure 2C, gene group no. 8).

**FIGURE 5:**
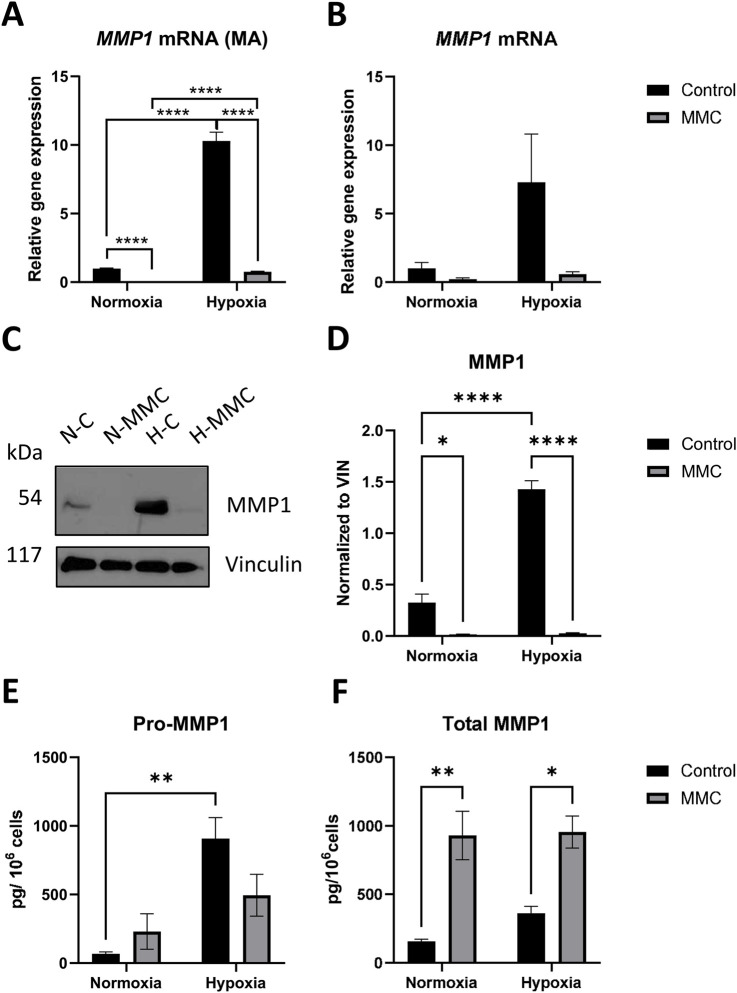
Expression of MMP1 on gene and protein levels upon treatment with mitomycin C (MMC) of SKOV3 cells in normoxia and hypoxia. MMP1 gene expression was measured using **(A)** HTA 2.0 microarrays and **(B)** RT-qPCR [normalized to PPIA and GUSB levels and normoxia control (NC) as 1]. **(C)** Immunoblot of MMP 1 protein (with vinculin as loading control). **(D)** Quantification of MMP1 protein levels by densitometry. **(E)** Secretion of pro-MMP1 protein levels measured by ELISA*.* **(F)** Secretion of total MMP1 protein levels measured by ELISA. **(B,D)** Results were normalized to normoxia control as 1. ****p < 0.0001, ***p < 0.001, **p < 0.01, and *p < 0.05; VIN, vinculin. A detailed statistics description is provided in the Supplementary Table S2.

*MMP1* expression was upregulated under hypoxia (*p* < 0.0001), while MMC treatment reduced *MMP1* under both pO_2_ conditions (p < 0.0001 in both pO_2_). Hypoxia-dependent induction of *MMP1* is abolished by MMC treatment (*p* < 0.0001, Figure 5A). Similar trends in *MMP1* gene expression in hypoxia were observed by RT-qPCR (H-C vs N-C: *p* = 0.1250; H-MMC vs H-C *p* = 0.1074, Figure 5B). MMP1 protein expression profile, assessed by Western blotting (WB), closely resembled the microarray analysis pattern (H-C vs N-C: *p* < 0.0001; N-MMC vs N-C: *p* = 0.0109; H-MMC vs H-C: *p* < 0.0001; Figures 5C,D). ELISA showed that the secreted pro-MMP1 (inactive form) significantly increased upon hypoxia (p = 0.0064) and tended to be downregulated by MMC (*p* = 0.1687, Figure 5E). Figure 5F shows total secreted MMP1 (including active and inactive forms of MMP1), which was significantly increased by MMC treatment under both normoxia (p = 0.0047) and hypoxia (*p* = 0.0211), in contrast to pro-MMP1. Pro- and total secreted MMP1 were also measured in three additional OC cell lines (Supplementary Figure S6), showing variable levels of induction either by MMC or hypoxia. The profiles of expression of the two forms of MMP1 under the influence of pO_2_ and MMC (Supplementary Figure S6) indicate that the four OC cell lines react similarly.

### Hypoxia and mitomycin C differentially shape the molecular landscape of genes as revealed by ingenuity pathway analysis

**FIGURE 6:**
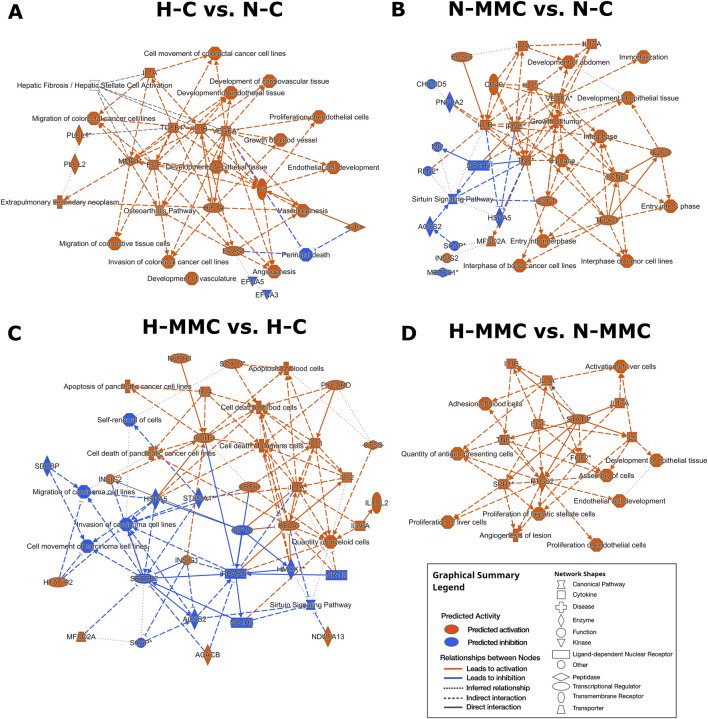
Graphical summary of the influence of mitomycin C (MMC) and hypoxia on SKOV3 ovarian cancer cells, generated using IPA (Ingenuity Pathway) Core Analysis, which selects and connects significant entities based on their p < 0.05. The summary includes canonical pathways, upstream regulators, diseases, and biological functions. Orange indicates the activation of the entity, and blue indicates the inhibition of the entity. **(A)** Effect of hypoxia (H-C vs. N-C), **(B)** effect of MMC in normoxia (N-MMC vs. N-C), **(C)** effect of MMC in hypoxia (H-MMC vs. H-C), and **(D)** effect of pO_2_ on MMC treatment (H-MMC vs. N-MMC).

To overview the main biological themes in the transcriptomic data, we performed pathway analysis using IPA software. It additionally allowed us to identify canonical pathways, upstream regulators, diseases, and biological functions. It also presents their relationships and infers relationships between previously unrelated entities. A graphical summary is shown in Figure 6. The effect of hypoxia (Figure 6A) was predicted using IPA to be mainly influenced by the activation of *HIF1A*, *VEGFA*, *IL1A*, *IL1B*, *TGFB1*, *F3*, *F10*, *PLCL1*, *PLCL2*, *MMP1*, *EPAS1*, and *EGF* as upstream regulators responsible for the gene expression pattern. The processes predicted to be mostly intensified are related to the development of endothelial cells and vasculature. Hypoxia also accelerates migration and invasion processes. The only genes predicted to be inhibited were *EFNA3* and *EFNA5*. When MMC treatment was performed under normoxic conditions (Figure 6B), IPA predicted the activation of *VEGFA*, *CD40*, *CCNE1*, *ECSIT*, *E2F1*, *E2F3*, *IFNG*, *IL1A*, *IL1B*, *IL15*, *IL17A*, *TNF*, and *TFDP1*. The processes predicted to be activated were cell cycle-related (S-phase entry and interphase). Other activated processes included tumor growth and cell immortalization. *ACSS2*, *CHCHD5*, *HSPA5 Mt2*, *MBTPS1*, *NR1H3*, *PNPLA2*, *RLN2*, and *SCAP* were predicted as inhibited. The inhibited pathway was sirtuin signaling. Under hypoxic conditions (Figure 6C), MMC treatment-activated upstream regulators predicted by IPA were *ACAB*, *CTSS*, *DDIT3*, *HTATIP2*, *IL1A*, *IL1RL2*, *IL18*, *IL24*, *IL32*, *IL36A*, *IRF6*, *MFSD2A*, *NDUFA13*, *NUPR1*, *PYCARD*, *RELA*, and *SOX17*. IPA predicted the inhibition of ACSS2, *HIF1A*, HMOX1, HSPA5, *NR1I2*, *NR1H3, PPARG*, SCAP, SDCBP, *SREBF2*, and *ST8SIA*. IPA predicted enhancement of blood and immune cell death, whereas cell movement and invasion were predicted to be reduced. Hypoxia during MMC treatment (Figure 6D) resulted in the predicted activation of *EGF*, *FGF2*, *IL1A*, *IL1B*, *IL6*, *IL17A*, *PTGS2*, *TNF*, *SPP1*, and *STAT3*. Among the genes that were predicted by IPA to be activated/inhibited upon MMC treatment, some presented altered transcription within the HTA microarray data (Supplementary Table S3). The enhanced processes were related to the development of epithelial tissue and endothelial cells, the proliferation of endothelial cells, liver and hepatic stellate cells, activation of liver cells, angiogenesis in lesions, cell assembly, blood cell adhesion, and an increase in antigen-presenting cells.

**FIGURE 7:**
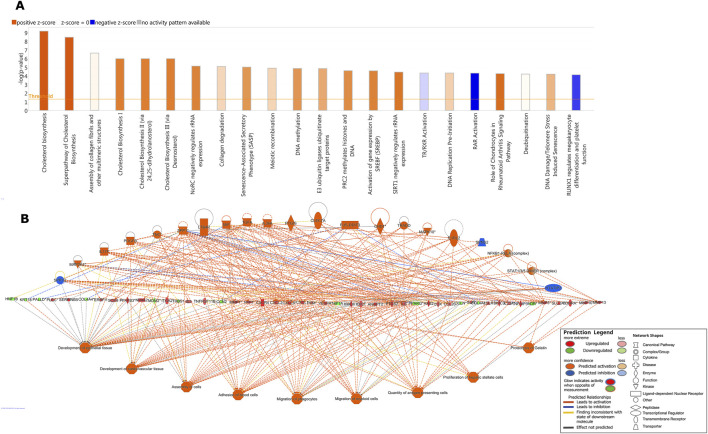
Ingenuity Pathway Analysis (IPA)-identified **(A)** key canonical enriched pathways. Y-axis represents the negative log of the p-value, indicating the significance of each canonical pathway. Bars are colored to indicate predicted pathway activity: orange for activation and blue for inhibition. **(B)** Upstream regulators that affected gene expression and effector pathways/processes when SKOV3 cells were treated with MMC in hypoxia (compared to N-MMC).

To further explore the pO_2_ effect on MMC treatment of SKOV3 cells at the intracellular level, we focused on activated and inhibited pathways identified by IPA. The highest z-score (indicating the activation of a given pathway, Figure 7A) was calculated for pathways related to cholesterol biosynthesis, followed by the negative regulation of rRNA by NoRC and the senescence-associated secretory phenotype (SASP), and methylation-related processes. The most inhibited pathways were RAR-activation and RUNX-regulation of megakaryocyte differentiation and platelet function. Upstream regulator (factors causing the observed gene expression changes) analysis (Figure 7B) revealed a variety of genes, changing the number of vital cellular and intercellular functions. According to IPA’s prediction, the activated regulators were *SPP1*, *MAP2K4*, *IL17F*, *TNC*, *PDGFB*, *LTB4R*, *MIF*, *TGFA*, *IL36A*, *PTGIS*, *CLEC7A*, *US-DDIT3*, *CHD1*, *TRADD*, *MAPK10*, *ILRL2*, *NfkB1-RelA*, and *STAT1/3/5* dimers. *HTATIP2*, *SPNS2*, and *SPRY2* were predicted to be inhibited. The effector genes with increased expression were *PLEC*, *ERRF1*, *SOX9*, *PFKF311*, *NR4A2*, *ITGA2*, *ID2*, *SERPINE*, *RSF*, *CXCR4, CXCL2*, *CXCL8*, *CXCL1*, *HMOX1*, *MMP1*, *MMP2*, *IL6ST*, *ANGPT2*, *PKM*, *VEGFA*, *IL6*, *PTGS2*, *HAS2*, *COX HAS2*, *CCL20*, *SLP1*, *LDLR*, *PIRC3*, *IL23A*, *TNFAIP3*, *MMP3*, *MMP13*, *SLC2A1E*, *EDNRA*, and *MMP10*. Downregulated genes included *GNF1B*, *KRT18*, *PALLD*, *COL4A4*, *MDM2*, *CCN2*, *THBS1*, *HIF1A*, *PPARG*, *OCLN*, *COL4A1*, and *VDR*. The predicted activation concerned the development of epithelial and cardiovascular tissues, cell assembly, adhesion of blood cells, migration of phagocytes and myeloid cells, quantity of APCs, tumor angiogenesis, proliferation of hepatic stellate cells, and proteolysis of gelatin.

## Discussion

To date, the effects of MMC on SKOV3 cells have been studied under standard conditions, i.e., normoxia. We analyzed the (transcriptomic) effects of MMC under hypoxic conditions, which resemble the *in vivo* tumor-growing conditions. Our study examines the global transcriptome modifications in SKOV3 OC cells following MMC treatment in hypoxia compared to normoxia. Therefore, chemotherapy with MMC in OC, like other drugs, may offer limited benefits unless the hypoxic microenvironment is first controlled.

MMC was more cytotoxic toward several types of cells maintained in hypoxia (Teicher and Sartorelli, 1980; Stratford and Stephens, 1989; Mistry et al., 2017). However, in many cases, hypoxia increased the resistance of tumor cells to drugs, e.g., cisplatin and paclitaxel (Klemba et al., 2020). We observed higher cytotoxicity of MMC toward hypoxic SKOV3, TOV112D, ES-2, and A2780 cells (Supplementary Figure S7), but hypoxia increased the IC_50_ values of MMC for two OC cell lines, namely, A2780 and ES2 (Supplementary Figure S1).

To identify the molecular changes due to hypoxia in SKOV3 cells and gain insight into the transcriptomic changes after MMC treatment, we performed analysis using HTA 2.0 microarrays. MMC was responsible for most of the observed changes in gene expression under both pO_2_ conditions. A total of 1,300 differentially expressed transcripts were commonly changed by MMC treatment in both pO_2_-values, but more than 1,000 gene changes were distinct and characteristic for each pO_2_ (Figure 2C). GO enrichment pathway analysis revealed that MMC treatment in normoxia affected BPs related to ribosome biogenesis and different types of RNA processing, which is consistent with previous findings on the mechanism of MMC action (Snodgrass et al., 2010). The preribosome was the most affected CC in normoxia but slightly altered in hypoxia, indicating an influence on protein maturation. Interestingly, hypoxic MMC treatment altered genes related to ECM properties (Figures 2D,E) (Gilkes et al., 2014), which is highly relevant for future translational applications of MMC. GO enrichment analysis also indicated changes upon hypoxic MMC treatment in cell–substrate junction and focal adhesion molecules (Ramteke et al., 2015). Other processes identified by GO analysis included DNA-binding transcription factor binding. Hypoxia additionally revealed changes in integrin functions, supporting ECM alterations by MMC. Therefore, hypoxia affects the response of OC cells to MMC by involving distinct molecular pathways from normoxia.

ECM-favored mobility and migration of hypoxic cancer cells depend on VEGF, the HIF-1 downstream transcription product and main modulator of angiogenesis. The modulation of VEGF gene expression and protein secretion (Figures 4, Figure 6A), along with the antiangiogenic effects of MMC (Gu et al., 2013; Li et al., 2015), confirms the key effect of pO_2_ in determining drug effectiveness.

Moreover, KEGG analysis identified cell senescence as the most significantly altered process in normoxia-MMC treatment, appearing less significant in hypoxia-MMC. MMC treatment affected cell morphology, notably including hallmark features of senescence, as evidenced by increased cell size and granularity (Goy et al., 2023). Different genes being involved under both oxygen conditions indicate distinct pathway activation (cell division in normoxia-MMC vs adenine transport in hypoxia-MMC), leading to the initiation of senescence (Zeng et al., 2018), which is important since senescence escape is favored by hypoxia (Olszewska et al., 2021).

Hypoxia-MMC treatment effects, analyzed by KEGG, revealed the IL-17 signaling pathway (hsa04657) genes in SKOV3 cells. IL17A, elevated in OC, promotes migration and invasion (Bilska et al., 2020; Guo and Zhang, 2022). The predominantly upregulated gene of the IL-17 pathway, *MMP1*, was increased in a HIF1α-dependent way (Shin et al., 2015). Checking the intracellular and secreted forms of MMP1, i.e., pro-MMP1, and total MMP1, i.e., MMC, suppressed *MMP1* mRNA expression and blocked its upregulation upon hypoxia (Figure 5). This difference was confirmed at the protein level for MMP1, occurring at the level of secreted proteins; under hypoxia, pro-MMP1 and total secreted form were not suppressed by the drug and even induced by MMC under both conditions. This indicates that the proportions of the active and pro-forms of the enzyme changed. This is important for the potential MMP1 activity in the hypoxic ECM, where it may degrade components like collagen and contribute to metastasis; this was confirmed in other OC cells, where pro- and total MMP1 proteins were upregulated by MMC and hypoxia (Supplementary Figure S6), consistent with previous observations (Shin et al., 2015; Belton et al., 2016; Pal-Ghosh et al., 2019). We report the effects of both factors on MMP1 forms, showing that OC cells may respond to hypoxia (TOV112D), while others were sensitive to MMC (A2780 and ES-2). MMP1 secretion upon hypoxia is redox-dependent (Shin et al., 2015). OC cell lines here (Supplementary Figure S6) show that MMC effects on both forms of the secreted protein are annihilated by hypoxia, proving that although the drug treatment and tumor microenvironment (TME; hypoxia) are activators of MMP1, their action can counteract one another.

Relationships between genes and biological processes uncovered previously unrecognized gene connections and pathways/processes by IPA. When pO_2_ was the only difference between MMC treatments of SKOV3 cells, the IPA canonical pathway analysis revealed strong activation of cholesterol biosynthesis and related pathways (all with *z*-score >5) (Figure 7A). Cholesterol biosynthesis dysregulation, often observed in OC patients, contributes to disease progression (He et al., 2021; Qusairy et al., 2023). *SPP1*, a gene encoding osteopontin, was among the identified genes recently shown to be important in cholesterol metabolism (Han et al., 2023). SPP1 levels correlated with clinical parameters and immune response in OC patients (Gao et al., 2022).

## Conclusion

Focusing on the data differences between *in vitro* and *in vivo-like* assays results under different TME conditions, this evidences highlights the influence of oxygen availability on cell responses and proves that the lack of consideration of hypoxia as a major regulator leads to data misinterpretation for translational applications.

Investigating the effect of MMC, an antibiotic relatively rarely used in OC therapy, on SKOV3 cells under normoxic and hypoxic conditions changed cell growth, morphology, number, LDH level, Ki67+ population, and cell cycle distribution. MMC- and pO_2_-induced transcriptomic changes were deciphered using HTA 2.0 microarrays. This combined treatment particularly highlighted the effect of pO_2_ modification on MMC activity. The most significant data were verified in three other OC cell lines, namely, TOV112D, ES-2, and A2780.

In normoxia, MMC mainly affected several ribosome-related pathways, while it modified the ECM in hypoxia, with *MMP1* as the most significantly upregulated gene and *SPP1* as a fundamental molecular regulator of processes, including cholesterol biosynthesis. Thus, the hypoxia-modified effects of MMC on OC cells uncovered therapeutic target genes to optimize MMC treatment efficacy.

Our data could also contribute to the development of OC cell virtual models that predict cell susceptibility to treatment (Phillips et al., 2000; Molinelli et al., 2013; Kozlowska et al., 2018). Our studies, therefore, open new perspectives in understanding the physiology of OC and will contribute to new models for the development of more effective therapeutic strategies.

## Data Availability

The datasets presented in this study are available at GEO repository, accession number GSE259424 (https://www.ncbi.nlm.nih.gov/geo/query/acc.cgi?&acc=GSE259424).

## Abbreviations

DEG, differentially expressed gene; ECM, extracellular matrix; GO, Gene Ontology; IPA, Ingenuity Pathway Analysis; KEGG, Kyoto Encyclopedia of Genes and Genomes; MMC, mitomycin C; OC, ovarian cancer; pO_2,_ oxygen tension; TME, tumor microenvironment.
