# Supplementary material for: Hypoxia alters the response of ovarian cancer cells to the mitomycin C drug
Source: Front Cell Dev Biol. 2025 Jun 13;13:1575134. doi: 10.3389/fcell.2025.1575134 (PMC12202450; doi:10.3389/fcell.2025.1575134)
Supplement: Supplementary file 1 [file DataSheet2.pdf]

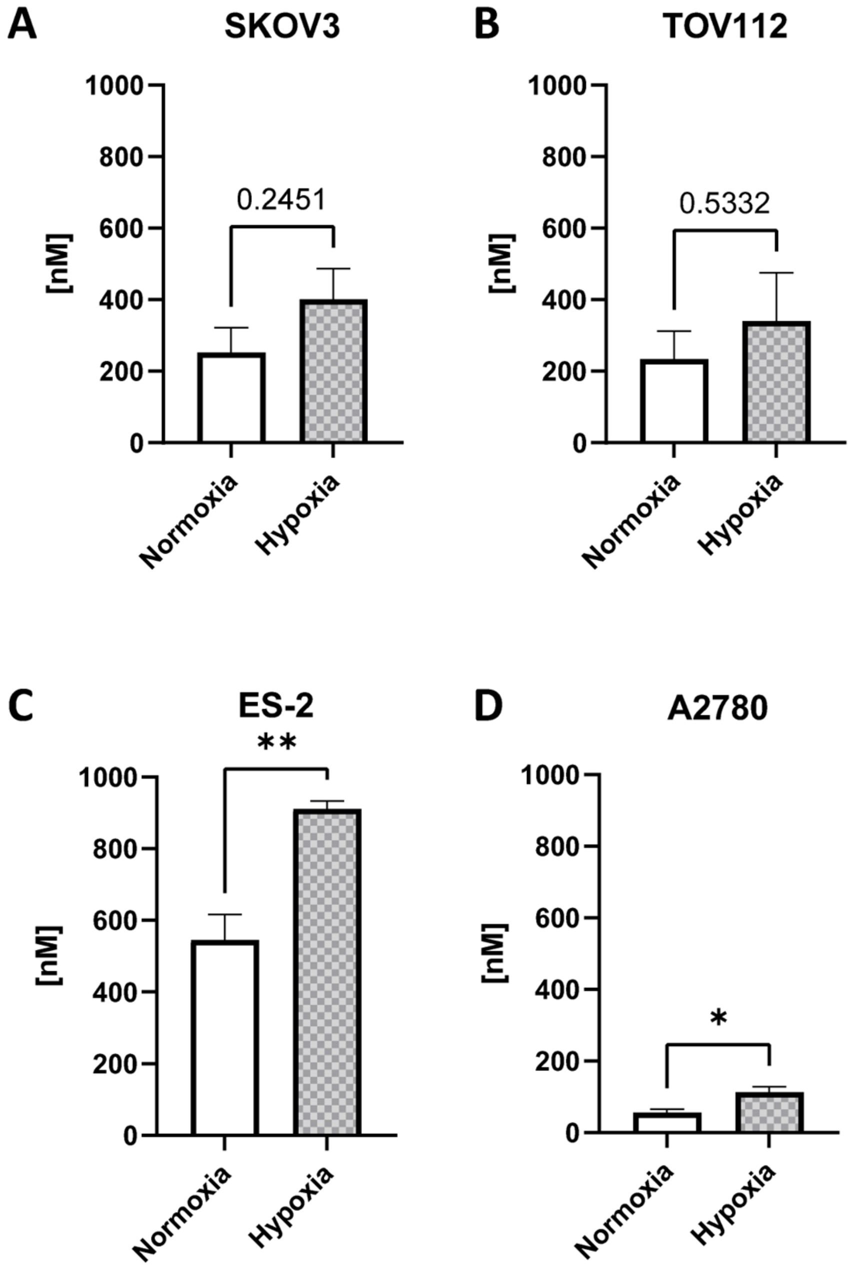

Supplementary Figure S1 IC_{50} values for ovarian cancer cell lines treated by mitomycin C in normoxia and hypoxia, evaluated by alamarBlue assay: **A** SKOV3 cells, **B** TOV112D cells, **C** ES-2 cells, **D** A2780 cells. A detailed statistics description is provided in the Supplementary Table S2.

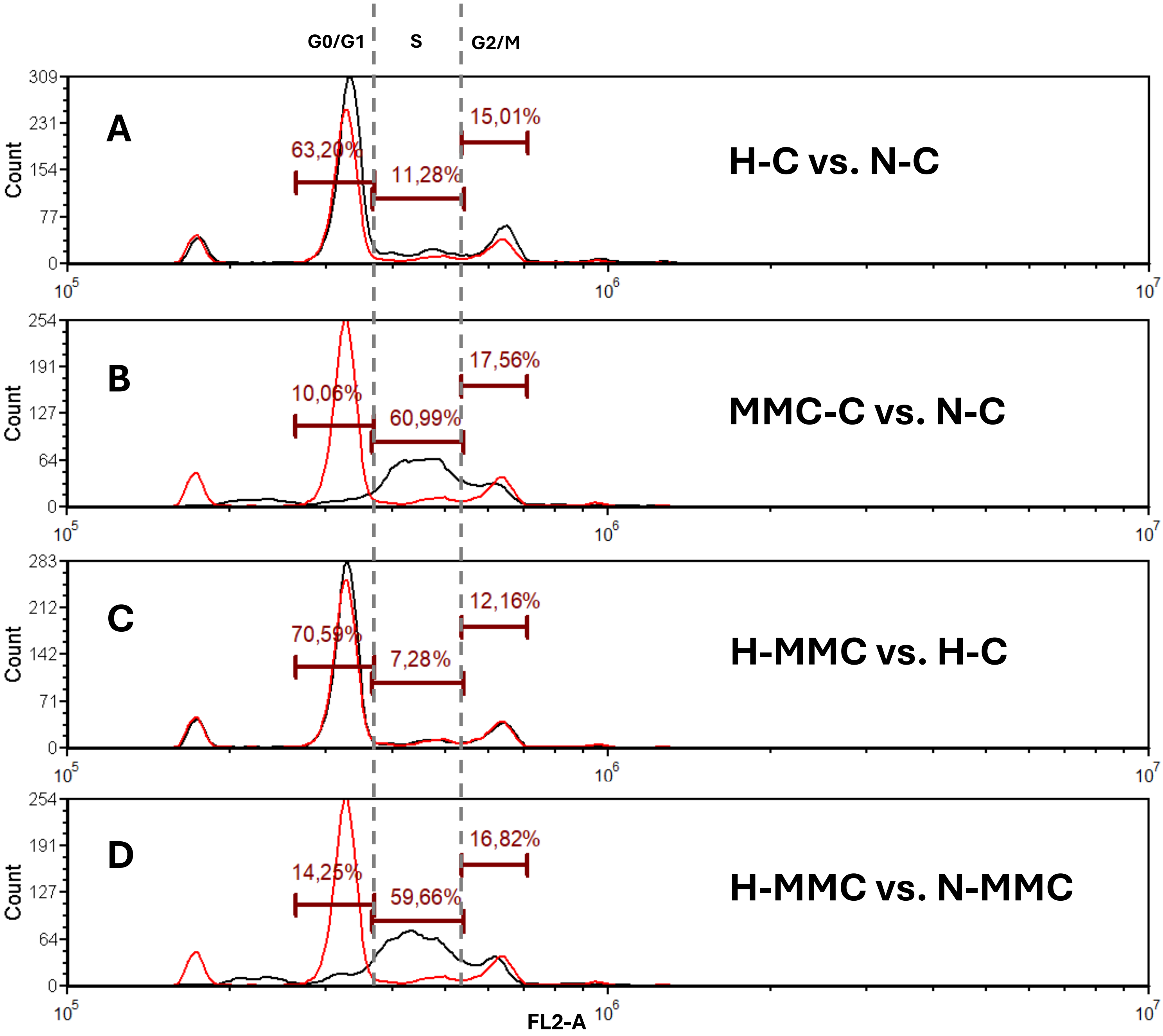

Supplementary Figure S2 Exemplary FACS flow cytometry histograms from cell cycle analysis of SKOV3 ovarian cancer cells treated with mitomycin C (MMC) in normoxia and hypoxia, labeled by propidium iodide. **A** The effect of hypoxia (H-C vs. N-C). **B** The effect of MMC in normoxia (N-MMC vs. N-C). **C** The effect of MMC in hypoxia (H-MMC vs. H-C). **D** The effect of pO_2 on MMC treatment (H-MMC vs. N-MMC). Red line indicates untreated control cells, black line tested sample.

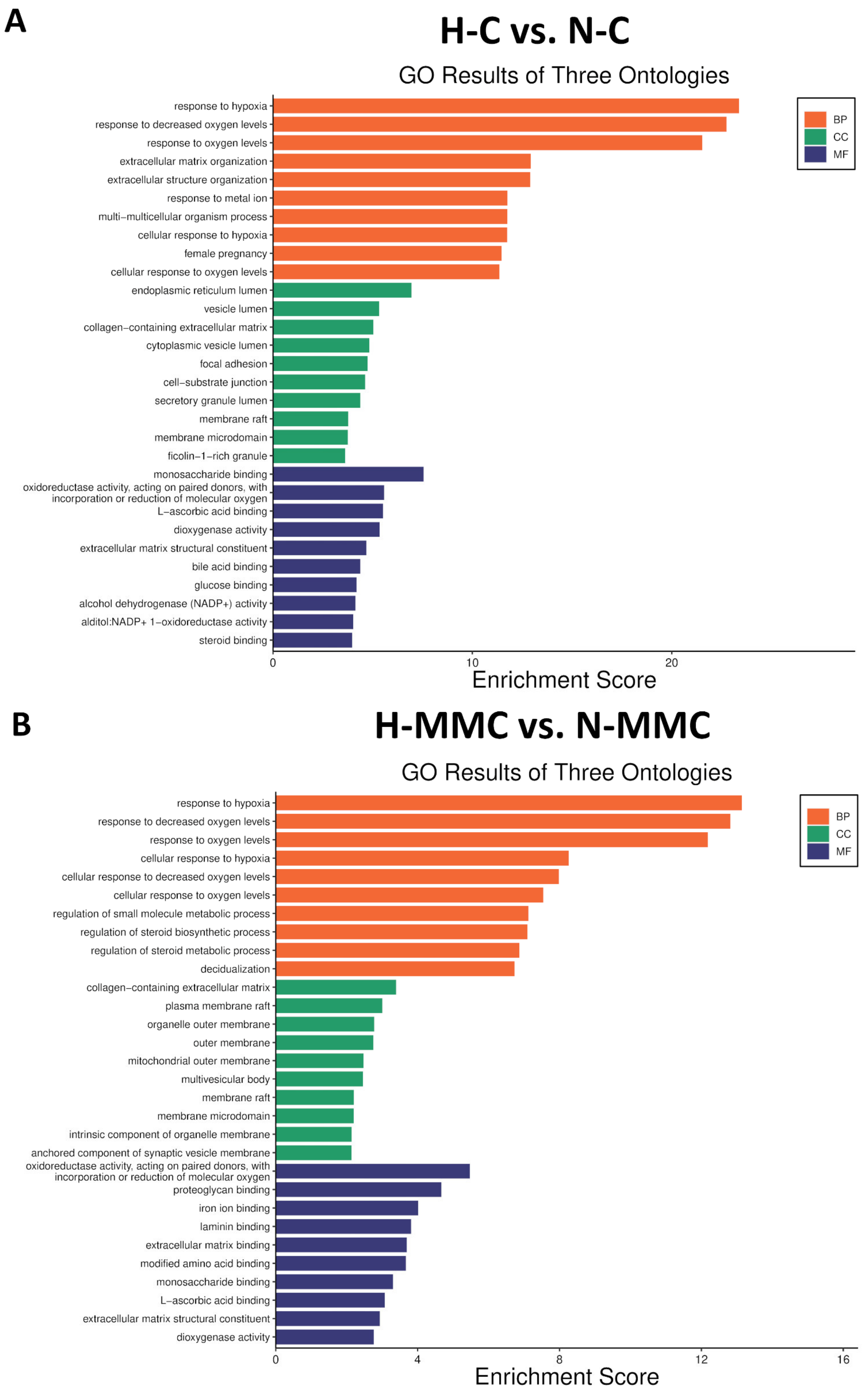

Supplementary Figure S3 The most significantly enriched gene ontologies (GO) showing the influence of hypoxia and MMC on SKOV3 cells, based on molecular functions (MF), biological processes (BP), and cellular components (CC). Enrichment score was calculated as -log(p-value). Fold change (FC) ≤ -2 and ≥ 2, $p<0.05$, genes without Entrez Gene ID were excluded from the analysis **A** H-C vs. N-C. **B** H-MMC vs. N-MMC.

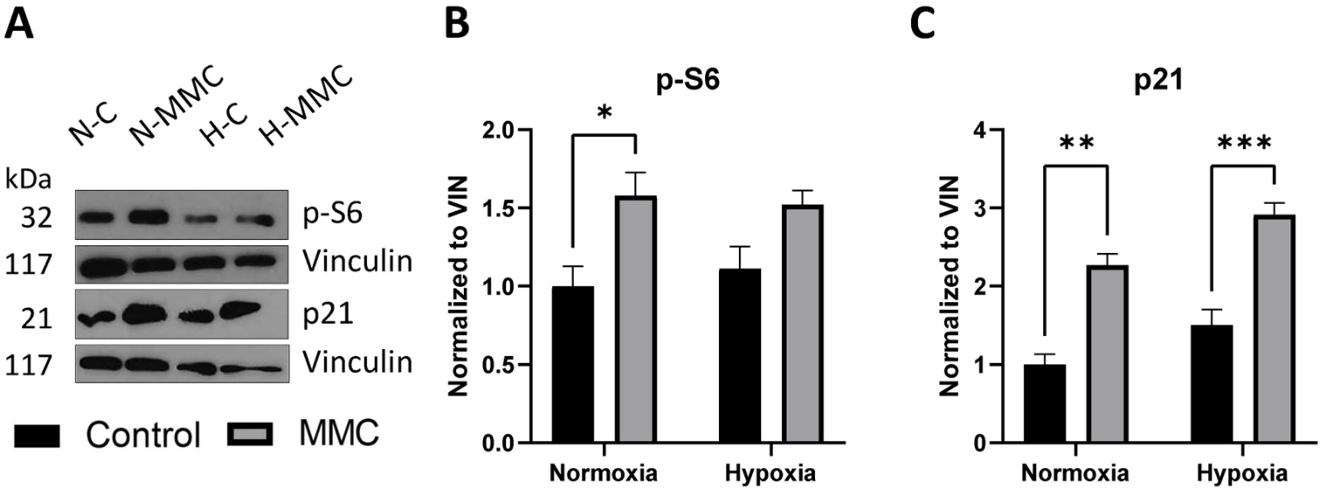

Supplementary Figure S4 Modifications of senescence features in SKOV-3 cells upon MMC treatment. **A** Representative western blots analysis showing expression of p-S6, p21 and Vinculin. Quantification of results was performed using densitometry with ImageJ software that shows ratio of certain protein to Vinculin (VIN) for **B** p-S6 and **C** p21. A detailed statistics description is provided in the Supplementary Table S2.

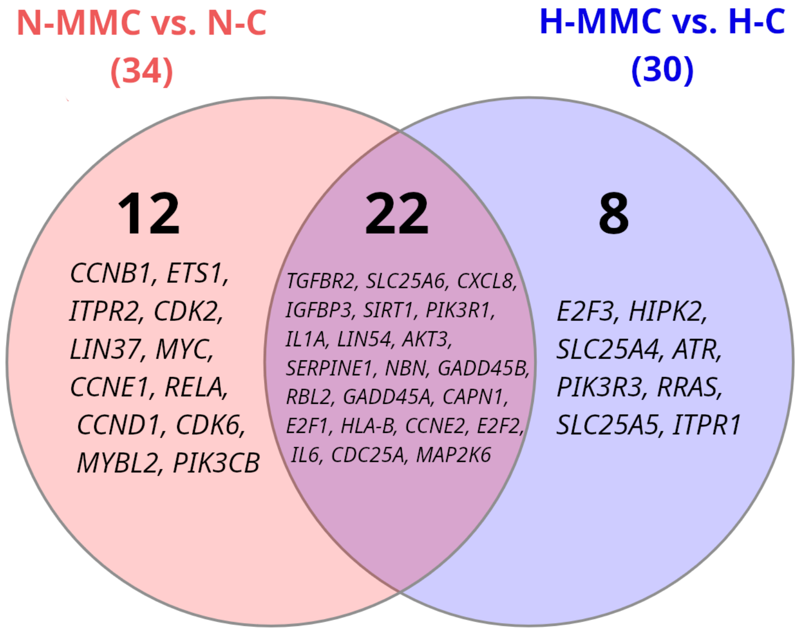

Supplementary Figure S5 Venn diagram of senescence-related differentially expressed genes under normoxic and hypoxic MMC treatment of SKOV3 cell.

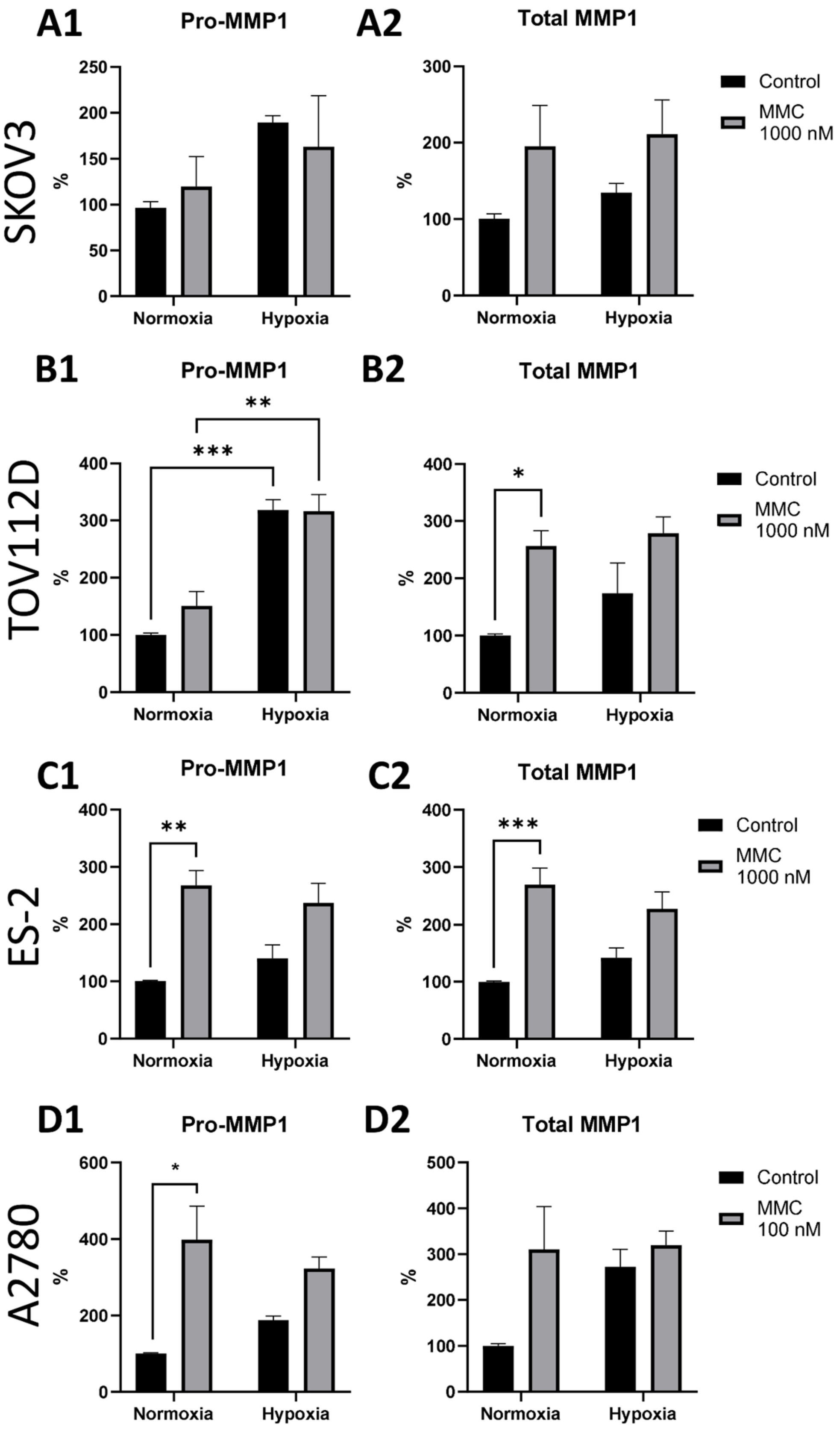

Supplementary Figure S6 Pro-MMP1 and total MMP1 secretion by OC cell lines treated with MMC in normoxia and hypoxia evaluated by ELISA, normalized to Alamar Blue value and expressed as the percentage of normoxic cells. **A** SKOV3 cells, **B** TOV112D cells, **C** ES-2, **D** A2780 cells. A detailed statistics description is provided in the Supplementary Table S2.

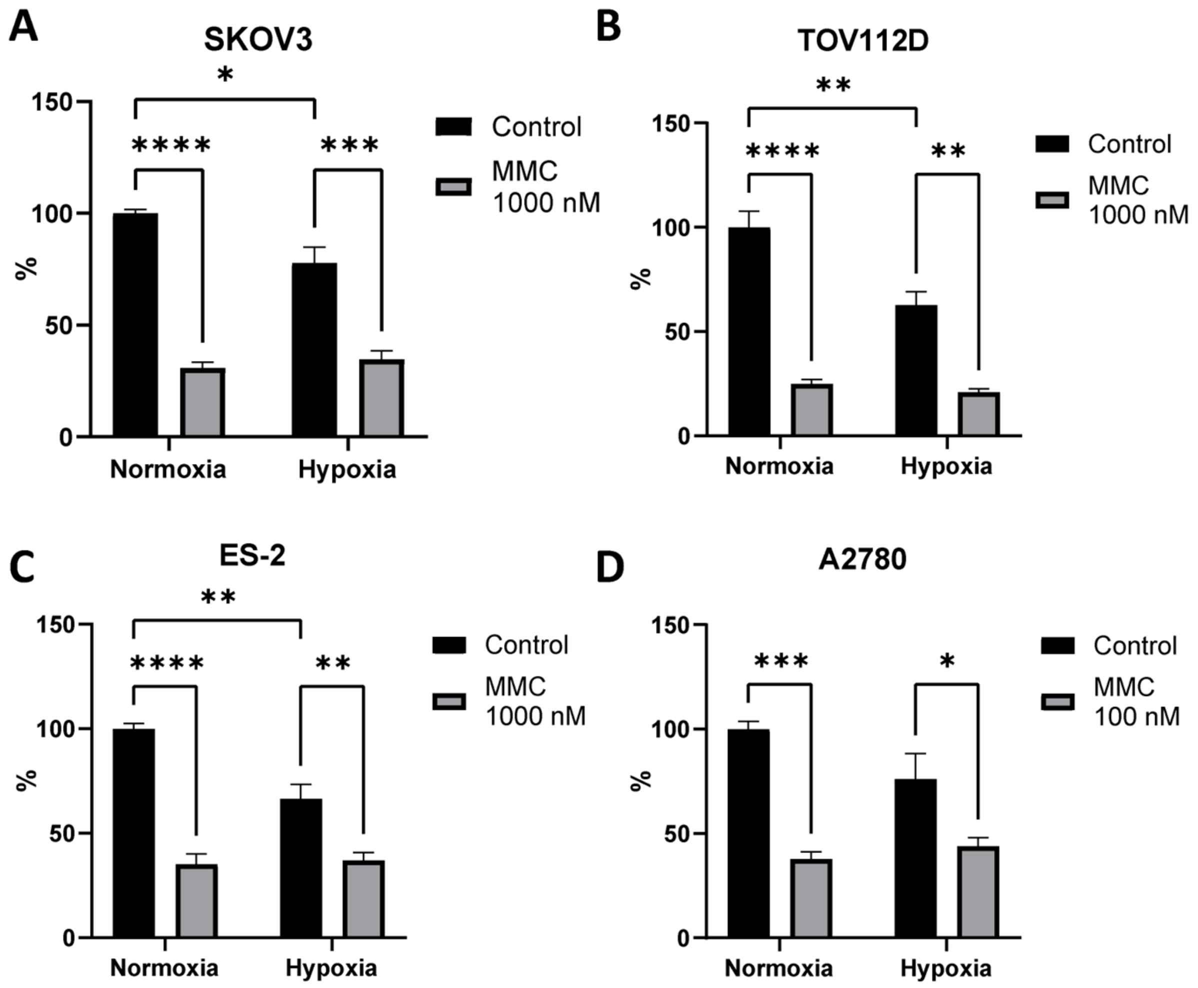

Supplementary Figure S7 Cell viability at a given dose (1000 nM or 100 nM) of MMC evaluated by Alamar Blue reaction in normoxia and hypoxia: **A** SKOV3 cells, **B** TOV112D cells, **C** ES-2 cells, **D** A2780 cells. A detailed statistics description is provided in the Supplementary Table S2.
