# Supplementary figures and images for: Hypoxia alters the response of ovarian cancer cells to the mitomycin C drug
Source: Front Cell Dev Biol. 2025 Jun 13;13:1575134. doi: 10.3389/fcell.2025.1575134 (PMC12202450; doi:10.3389/fcell.2025.1575134)

# Western blots for MMP1, raw unprocessed blots

## Repetitions 1 and 2

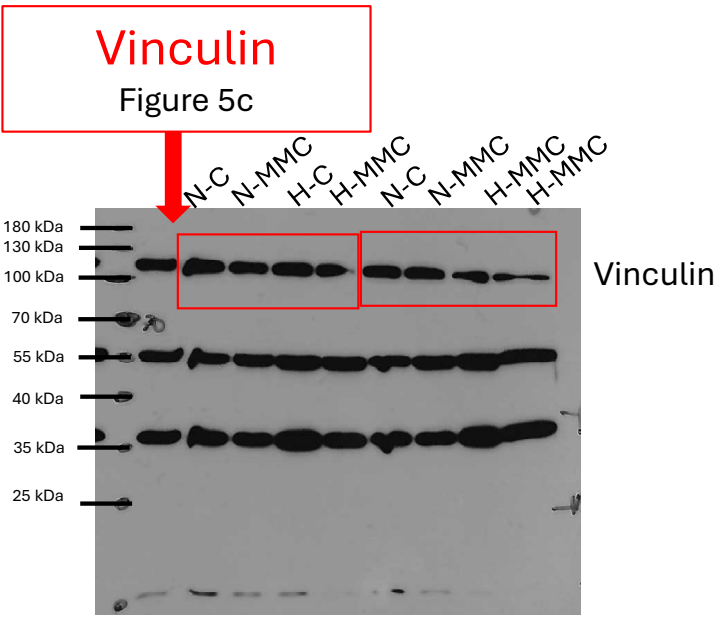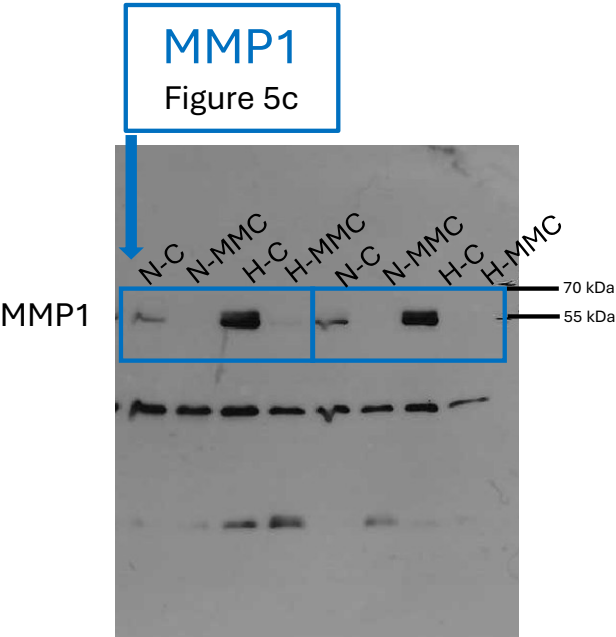

## Repetition 3

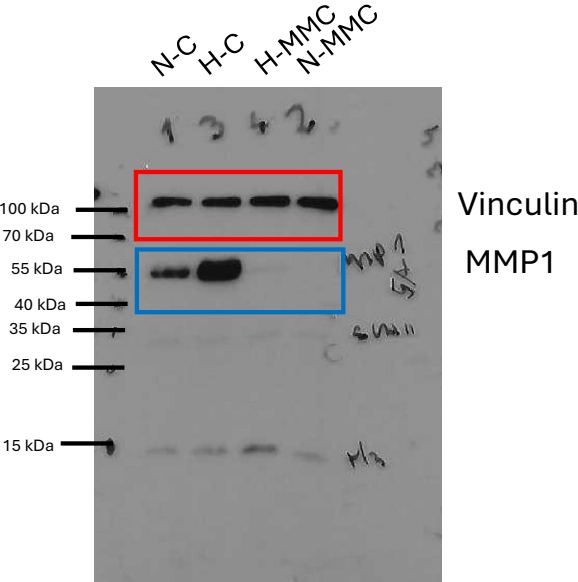

## Repetition 4

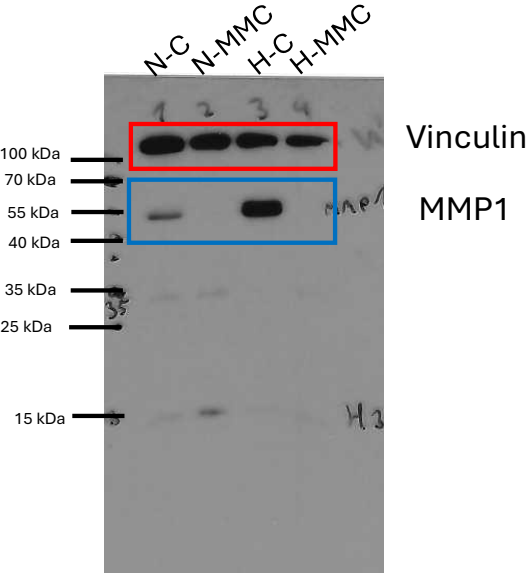

Supplement: Supplementary file 3 [file DataSheet3.pdf]
