# Supplementary material for: Hypoxia alters the response of ovarian cancer cells to the mitomycin C drug
Source: Front Cell Dev Biol. 2025 Jun 13;13:1575134. doi: 10.3389/fcell.2025.1575134 (PMC12202450; doi:10.3389/fcell.2025.1575134)
Supplement: Supplementary file 4 [file Table2.docx]

**Supplementary Table S2. Statistics**

| **Fig.** | **Name** | **Effect of O_2_** | **Effect of MMc** | **Effect of interaction** | **H-C vs. N-C**  **p-value** | **N-MMC vs. N-C**  **p-value** | **H-MMC vs. H-C**  **p-value** | **H-MMC vs. N-MMC**  **p-value** |
| --- | --- | --- | --- | --- | --- | --- | --- | --- |
| **Figure 1** | | | | | | | | |
| **1A** | Cell size | F(1, 12) = 0.3243  p=0.5796 | F(1, 12) = 62.55  p<0.0001 | F(1, 12) = 0.01162  p=0.9160 | 0.9874 | 0.0005 | 0.0007 | 0.9623 |
| **1A** | Cell granularity | F(1, 12) = 0.9808  p=0.3416 | F(1, 12) = 180.7  p<0.0001 | F(1, 12) = 1.871  p=0.1965 | 0.3809 | < 0.0001 | < 0.0001 | 0.9930 |
| **1B** | Cell viability | F(1, 32) = 19.67  p=0.0001 | F(1, 32) = 792.5  p<0.0001 | F(1, 32) = 13.52  p=0.0009 | < 0.0001 | < 0.0001 | <0.0001 | 0.9496 |
| **1C** | LDH | F(1, 8) = 9.972  p=0.0134 | F(1, 8) = 261.9  p<0.0001 | F(1, 8) = 4.530  p=0.0660 | 0.8833 | < 0.0001 | < 0.0001 | 0.0237 |
| **1D** | Ki67+ population | F(1, 8) = 1.846  p=0.2113 | F(1, 8) = 33.59  p=0.0004 | F(1, 8) = 0.1613  p=0.6985 | 0.6185 | 0.0214 | 0.0101 | 0.9030 |
| **1E** | Phase G0/G1 | F(1, 8) = 1.988  p=0.1962 | F(1, 8) = 90,63  p<0,0001 | F(1, 8) = 0.02099  p=0.8884 | 0.8081 | 0.0006 | 0.0007 | 0.6997 |
| **1E** | Phase S | F(1, 8) = 0.2994  p=0.5992 | F(1, 8) = 58.47  p<0.0001 | F(1, 8) = 0.07869  p=0.7862 | 0.9338 | 0.0036 | 0.0023 | 0.9974 |
| **1E** | Phase G2/M | F(1, 8) = 0.6618  p=0.4395 | F(1, 8) = 5.898  p=0.0413 | F(1, 8) = 0.07099  p=0.7966 | 0.9790 | 0.2979 | 0.4653 | 0.8685 |
| **Figure 4** | | | | | | | | |
| **4A** | *VEGF* mRNA  (RT-qPCR) | F(1, 8) = 158.0  p<0.0001 | F(1,8) = 48.46  p=0.0001 | F(1, 8) = 49.08  p=0.0001 | <0.0001 | >0.9999 | <0.0001 | 0.0182 |
| **4B** | VEGF protein (ELISA) | F(1, 8) = 2.487  p=0.1534 | F(1, 8) = 3.333  p=0.1053 | F(1, 8) = 14.43  p=0.0052 | 0.0218 | 0.5355 | 0.0172 | 0.4441 |
| **Figure 5** | | | | | | | | |
| **5A** | *MMP1* mRNA (MA) | Condition FDR F-test p<0.0001 | | | <0.0001 | <0.0001 | <0.0001 | <0.0001 |
| **5B** | *MMP1* mRNA  (RT-qPCR) | F(1, 8) = 3.492  p=0.0986 | F(1, 8) = 4.413  p=0.0689 | F(1, 8) = 2.763  p=0.1351 | 0.1350 | 0.9889 | 0.1074 | 0.9988 |
| **5D** | MMP1 protein (WB) | F(1, 17) = 79.55  p<0.0001 | F(1, 17) = 188.0  p<0.0001 | F(1, 17) = 76.47  p<0.0001 | <0.0001 | 0.0109 | <0.0001 | 0.9993 |
| **5E** | Pro-MMP1 protein (ELISA) | F(1, 8) = 19.43  p=0.0023 | F(1, 8) = 1.007  p=0.3449 | F(1, 8) = 5.288  p=0.0505 | 0.0064 | 0.7973 | 0.1687 | 0.4846 |
| **5F** | MMP1 Total protein (ELISA) | F(1, 8) = 1.110  p=0.3229 | F(1, 8) = 38.76  p=0.0003 | F(1, 8) = 0.6712  p=0.4364 | 0.5743 | 0.0047 | 0.0211 | 0.9982 |
| \| **Supplementary Figure S1** \| \| \| \| \| --- \| --- \| --- \| --- \| \| **Fig.** \| **Name** \| **H vs. N p-value** \| **t, df** \| \| **S1A** \| SKOV3 \| 0.2451 \| t=1.361, df=4 \| \| **S1B** \| TOV112D \| 0.5332 \| t=0.6811, df=4 \| \| **S1C** \| ES-2 \| 0.0027 \| t=4.922, df=6 \| \| **S1D** \| A2780 \| 0.0169 \| t=3.278, df=6 \| | | | | | | | | |
| **Fig.** | **Name** | **Effect of O_2_** | **Effect of MMC** | **Effect of interaction** | **H-C vs. N-C**  **p-value** | **N-MMC vs. N-C**  **p-value** | **H-MMC vs. H-C**  **p-value** | **H-MMC vs. N-MMC**  **p-value** |
| **Supplementary Figure S4** | | | | | | | | |
| **S4A** | p-S6 protein level | F(1, 12) = 0.04693  p=0.8321 | F(1, 12) = 15.01  p=0.0022 | F(1, 12) = 0.4611  p=0.5100 | 0.9193 | 0.0323 | 0.1627 | 0.9873 |
| **S4B** | P21 protein level | F(1, 8) = 13.75  p=0.0060 | F(1, 8) = 73.93  p<0.0001 | F(1, 8) = 0.2035  p=0.6639 | 0.1764 | 0.0019 | 0.0009 | 0.0723 |
| **Supplementary Figure S6** | | | | | | | | |
| **S6**  **A1** | SKOV3  Pro-MMP1 | F(1, 8) = 4.383  p=0.0696 | F(1, 8) = 0.002607  p=0.9605 | F(1, 8) = 0.5867  p=0.4657 | 0.2566 | 0.9555 | 0.9360 | 0.7858 |
| **S6**  **A2** | SKOV3  Total MMP1 | F(1, 8) = 0.4910  p=0.5034 | F(1, 8) = 5.765  p=0.0431 | F(1, 8) = 0.06363  p=0.8072 | 0.9041 | 0.3091 | 0.9881 | 0.4701 |
| **S6**  **B1** | TOV112D  Pro-MMP1 | F(1, 8) = 80.63  p<0.0001 | F(1, 8) = 1.264  p=0.2935 | F(1, 8) = 1.522  p=0.2523 | 0.0004 | 0.3980 | 0.9998 | 0.0026 |
| **S6**  **B2** | TOV112D  Total MMP1 | F(1, 8) = 2.132  p=0.1824 | F(1, 8) = 15.72  p=0.0042 | F(1, 8) = 0.6255  p=0.4518 | 0.4340 | 0.0399 | 0.1911 | 0.9629 |
| **S6**  **C1** | ES-2  Pro-MMP1 | F(1, 12) = 0.03942  p=0.8459 | F(1, 12) = 28.95  p=0.0002 | F(1, 12) = 2.105  p=0.1725 | 0.6580 | 0.0020 | 0.0692 | 0.8124 |
| **S6**  **C2** | ES-2  Total MMP1 | F(1, 12) = 0.0001233  p=0.9913 | F(1, 12) = 32.19  p=0.0001 | F(1, 12) = 3.563  p=0.0835 | 0.5555 | 0.0009 | 0.0823 | 0.5645 |
| **S6**  **D1** | A2780  Pro-MMP1 | H(4) = 12.73, p<0.0001 | | | >0.9999 | 0.0140 | 0.5258 | >0.9999 |
| **S6**  **D2** | A2780  Total MMP1 | F(1, 12) = 2.940  p=0.1121 | F(1, 12) = 5.950  p=0.0312 | F(1, 12) = 2.399  p=0.1474 | 0.1507 | 0.0646 | 0.9205 | 0.9994 |
| **Supplementary Figure S7** | | | | | | | | |
| **S7A** | SKOV3 viability | F(1, 8) = 4.501  p=0.0667 | F(1, 8) = 169.0  p<0.0001 | F(1, 8) = 9.287  p=0.0159 | 0.0266 | <0.0001 | 0.0005 | 0.9110 |
| **S7B** | TOV112D  Viability | F(1, 8) = 16.10  p=0.0039 | F(1, 8) = 128.3  p<0.0001 | F(1, 8) = 10.47  p=0.0120 | 0.0040 | <0.0001 | 0.0020 | 0.9442 |
| **S7C** | ES-2  Viability | F(1, 12) = 10.90  p=0.0063 | F(1, 12) = 96.05  p<0.0001 | F(1, 12) = 13.44  p=0.0032 | 0.0017 | <0.0001 | 0.0046 | 0.9937 |
| **S7D** | A2780  Viability | F(1, 12) = 1.664  p=0.2214 | F(1, 12) = 46.90  p<0.0001 | F(1, 12) = 4.831  p=0.0483 | 0.1167 | 0.0002 | 0.0287 | 0.9162 |

Abbreviations:

H-C = Hypoxia control

N-C = normoxia control

N-MMC = normoxia with mitomycin C

H-MMC = hypoxia with mitomycin C

H = hypoxia (cells in hypoxia)

N = normoxia (cells in normoxia)
