# Supplementary material for: Hypoxia alters the response of ovarian cancer cells to the mitomycin C drug
Source: Front Cell Dev Biol. 2025 Jun 13;13:1575134. doi: 10.3389/fcell.2025.1575134 (PMC12202450; doi:10.3389/fcell.2025.1575134)
Supplement: Supplementary file 6 [file DataSheet1.docx]

**Supplementary Materials and methods**

***Cell line and treatment procedure***

Human ovarian cancer cell lines SKOV3 (ATCC, Cat#HTB-77), TOV112D (ATCC, Cat#CRL-11731), ES-2 (ATCC, Cat#CRL-1978) and A2780 (ECACC, Cat#93112519) cultured in OPTI MEM medium supplemented with 2% - FBS (Thermo Fisher Scientific, USA, OptiMEM: Cat#31985070, FBS: Cat#A5209401). Cells were passaged at 80% confluence by detaching with Accutase (Biolegend, USA, Cat#423201). Cells used in the experiments were Mycoplasma free (as tested by PromoKine, Cat#PK-CA91-1096) and did not exceed the 10^th^ passage.

Cells seeded (SKOV3 - 3000 cell/well, TOV112D - 4500 cells/well, ES-2 -3500 cells/well and A2780 - 5000 cells/well) in 96-well plate in 100µl of medium were incubated for 24h in normoxic conditions. After 24h medium was changed to preconditioned medium (incubated in normoxia or hypoxia for 24h before the experiment), with or without range of mitomycin C (MMC) dose: 1nM, 10 nM, 100 nM, 1000 nM and 10000 nM and the flasks were incubated for the following 72h. Then supernatants were collected and alamarBlue (Thermo Fisher Scientific, USA, Cat#DAL1025) test allowing to assess MMC cytotoxicity was performed on the cells remaining in the well (according to manufacturer protocol). Supernatants were later used for ELISA assessment of pro-MMP1 and total MMP1 (DuoSet ELISA kits, R&D Systems, USA, pro-MMP1: Cat#DY900-05, total MMP1: Cat#DY901B) secretion levels as described in materials and methods section. The ELISA results were normalized to alamarBlue value corresponding to the dose of MMC treatment to standardize for the number of cells in the well.
